# Adaptation, Plasticity, and Extinction in a Changing Environment: Towards a Predictive Theory

**DOI:** 10.1371/journal.pbio.1000357

**Published:** 2010-04-27

**Authors:** Luis-Miguel Chevin, Russell Lande, Georgina M. Mace

**Affiliations:** 1 Division of Biology, Imperial College London, Silwood Park, United Kingdom; 2 Centre for Population Biology, Imperial College London, Silwood Park, United Kingdom; The University of North Carolina, United States of America

## Abstract

The authors analyze developmental, genetic, and demographic mechanisms by which populations tolerate changing environments and discuss empirical methods for determining the critical rate of sustained environmental change that causes population extinction.

## Summary

Many species are experiencing sustained environmental change mainly due to human activities. The unusual rate and extent of anthropogenic alterations of the environment may exceed the capacity of developmental, genetic, and demographic mechanisms that populations have evolved to deal with environmental change. To begin to understand the limits to population persistence, we present a simple evolutionary model for the critical rate of environmental change beyond which a population must decline and go extinct. We use this model to highlight the major determinants of extinction risk in a changing environment, and identify research needs for improved predictions based on projected changes in environmental variables. Two key parameters relating the environment to population biology have not yet received sufficient attention. Phenotypic plasticity, the direct influence of environment on the development of individual phenotypes, is increasingly considered an important component of phenotypic change in the wild and should be incorporated in models of population persistence. Environmental sensitivity of selection, the change in the optimum phenotype with the environment, still crucially needs empirical assessment. We use environmental tolerance curves and other examples of ecological and evolutionary responses to climate change to illustrate how these mechanistic approaches can be developed for predictive purposes.

## Introduction

Global climate change, over-exploitation, and habitat alteration are causing sustained and consistent pressures on wild populations [1]. Because of habitat fragmentation, many species will not be able to track their preferred environment in space, and must therefore adapt in situ to avoid extinction. What determines the maximum rate of environmental change that populations can cope with? Understanding this will inform both models and management plans about where critical thresholds lie or what might affect the potential resilience of particular species or ecological communities.

Two main approaches exist for studying the impact of environmental change on species persistence—niche modelling and mechanistic population modelling. On the one hand, “climate envelope models” (or “niche models”) are correlative and focused on the environment. Their conceptual background traces to Hutchinson's multidimensional representation of the niche [2]. They use measurements of environmental variables and records of species presence and absence to infer abiotic correlates of the realized niche of a species. By projecting this niche on a map with environmental data, the spatial locations that satisfy the basic requirements of the species are identified. Niche models have been combined with climate projections to predict range shifts and extinction rates [3]. Aside from the methodological caveats of this approach (changing covariance of environmental variables [4], population demography not in equilibrium with changing climate [5], spatial scale of the analysis [6],[7], and source-sink dynamics allowing individuals to exist outside their niche [8]), its major drawback is that it does not account for the biological processes underlying adaptation of a species to its environment. It cannot identify or incorporate the biological differences among species that determine whether or not they can persist in situ. While a new generation of “process-based” (or “mechanistic”) niche models is being developed to overcome these limitations [9]–[11], they currently rely on simplified demographic processes and generally overlook evolution in response to climate change (but see [12]). Niche modelling is thus currently more suited to understand changes in the distribution of suitable environments for a species at a continental scale than to predict population persistence or guide conservation plans.

On the other hand, mechanistic population modelling focuses on the biological processes that underlie population persistence. By combining evolutionary genetics and demography, the conditions that allow a population to maintain a positive intrinsic growth rate in the face of environmental change can be predicted theoretically [13],[14] and studied empirically [15]. The main focus of mechanistic population studies is not environmental variables, but ecologically important phenotypic traits (morphology, physiology, phenology, or behaviour) that affect population growth through their influence on life history. In contrast to niche modelling, this approach allows identification of factors that potentially limit population persistence in a changing environment. However, unless environmental variables are included, as well as phenotypic traits, this approach cannot be used to project the fate of populations.

Here, we propose a model of evolution and population growth to address how demographic and evolutionary constraints limit the persistence of a geographically isolated population under sustained environmental change. This model generalizes an earlier one by Lynch and Lande [13] by including phenotypic plasticity (see Box 1–Glossary) and an explicit environmental variable such as temperature. We use it to highlight the determinants of extinction risk and to identify research needs for a more predictive approach to population persistence in a changing environment. Research directed towards key determinants of extinction risk that are often overlooked in current empirical work may allow population biologists to take advantage of abundant environmental data in order to begin developing predictions about population persistence under alternative scenarios of climate change [16], which can help to guide conservation policies and management plans.

**Box 1. Glossary**

**critical rate of environmental change:** maximum rate of sustained environmental change that allows long-term persistence of a population, denoted as *η_c_*.

**environmental sensitivity of selection:** change in the optimum phenotype with the environment. For a linear relationship, it is measured by the slope *B*.

**generation time:** the average age of parents of a cohort of newborn individuals, denoted as *T*. With discrete non-overlapping generations, *T* is the mean time between successive reproductive episodes in the population.

**genetic variance:** the genetic component of phenotypic variance *σ* ^2^, or more precisely, “additive genetic variance” *h* ^2^ *σ* ^2^, the statistically additive component of phenotypic variance determining the resemblance between offspring and parents, and the genetic response to selection.

**heritability:** the proportion of phenotypic variance in a trait due to additive genetic effects, denoted as *h* ^2^.

**intrinsic rate of increase:** population growth rate in the absence of intra- or inter-specific competition. For a perfectly adapted population with mean phenotype at the optimum, the intrinsic rate of increase is denoted as *r_max_*.

**norm of reaction:** the expected phenotype of a given genotype as a function of the environment.

**phenotypic plasticity:** direct influence of the environment on individual phenotypes through developmental mechanisms. For a linear norm of reaction plasticity is measured by the slope *b*.

**quantitative trait:** continuously distributed phenotypic character, with phenotypic variance in a population determined by multiple polymorphic genes and environmental effects.

**stabilizing selection:** natural selection such that individual fitness decreases with increasing phenotypic deviation from an optimum. Its strength is measured by *γ*.

**tolerance curve:** fitness or performance as a function of the environment.

## Extinction Risk under Sustained Environmental Change

There are three mechanisms by which a population can persist when its local environment changes: dispersal to track its preferred environment in space, genetic evolution to the new local conditions, or phenotypic plasticity [17],[18]. Using the model in Box 2 and Text S1, we give an overview of the determinants of extinction risk for an isolated population with a restricted geographic range under sustained environmental change, assuming dispersal is not possible. We consider cases where all other possible factors influencing extinction risk (demographic and environmental stochasticity, inbreeding depression, genetic drift [19]) can be ignored relative to the impact of sustained environmental change. The determinants of extinction risk then belong to three main classes.

**Box 2. A Model of Phenotypic Evolution and Persistence under Sustained Environmental Change**

We assume that a continuous environmental parameter *ε* (e.g., temperature or precipitation) changes at a constant rate *η* in time. Adaptation to this changing environment is mediated by a quantitative trait *z* that determines fitness. Selection on multiple correlated traits also can be incorporated [20], but for simplicity we focus on single trait. Population growth is assumed to be density-independent, which can be a good approximation even with density dependence, if the population remains well below the carrying capacity because of maladaptation. In contrast to the original model and its recent extensions [13],[20], we include phenotypic plasticity in the trait *z*, such that a given change in the environment directly modifies the phenotype of each individual by a constant amount. The rate of environmental change is expressed per unit time (e.g., °C per year) instead of per generation.

Under sustained environmental change, assuming constant genetic and phenotypic variance and strength of stabilizing selection, the rate of phenotypic evolution eventually reaches a steady state where the mean phenotype lags a constant distance behind the changing optimum phenotype (Text S1). If this distance is such that the population has a negative growth rate, extinction occurs. The maximum rate of environmental change that allows long-term persistence of the population (or critical rate of environmental change) is
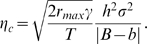
(1)

The original model [13] revealed how the phenotypic variance *σ* ^2^ and heritability *h* ^2^ of the trait, the strength of stabilizing selection *γ*, and the maximum intrinsic rate of population growth *r_max_* determined the critical rate of environmental change in the absence of phenotypic plasticity.

Three other parameters included here emphasize important factors affecting persistence when the directional environmental change is expressed in environmental units per unit time. First is the generation time *T*. The remaining two parameters are the *environmental sensitivity of selection B*, which measures how changes in the environment influence the optimum phenotype, and *phenotypic plasticity b*, which quantifies the direct impact of the environment on development of individual phenotypes. The critical rate of environmental change for long-term persistence increases with decreasing absolute difference between the environmental sensitivity of selection and phenotypic plasticity. Although plasticity causes weaker natural selection on the trait and smaller genetic response to selection, this is more than compensated by the plastic phenotypic change that brings the mean phenotype closer to the optimum (Text S1).

Plasticity may also entail a fitness cost [59]. The cost of plasticity decreases *r_max_*, which can then be formulated as
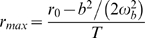
(2)where *r*_0_ is the per-generation growth rate of a population with optimum phenotype and no plasticity, and 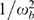 quantifies the magnitude of the cost for a given plasticity *b*. By decreasing *r_max_*, the cost of plasticity decreases the critical rate of environmental change, thus opposing the beneficial effect of plasticity on *η_c_*. The overall effect of phenotypic plasticity on the critical rate of environmental change is shown in Figure 2 for several values of the cost of plasticity. When the cost of plasticity is above a threshold (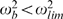), there is an intermediate value of plasticity that maximizes the critical rate of environmental change.

(1) *Demographic properties of the population*. These are components of the population growth rate that are not affected by adaptation to the changing environment. They include the generation time, *T*, and the maximum intrinsic rate of increase *r_max_* (per unit time) of a population with optimum mean phenotype. Even if these parameters are not affected by the environmental change, they do constrain the rate of adaptation. Populations with longer generations must evolve faster per generation to adapt to a given rate of environmental change, and populations with a low *r_max_* will reach extinction before they can adapt to rapid environmental change [13]–[15],[20]. The specific influence of each of these variables on extinction risk across taxa also depends on their covariation. Broad-scale studies of life-history traits indicate that the per-generation intrinsic rate of increase is of the same order of magnitude across several taxa, whereas *r_max_* per unit time is roughly inversely proportional to the generation time [21]–[23]. As a consequence, the critical rate of environmental change should be generally lower for species with longer generation times, which will be at greater extinction risk.

(2) *Evolutionary potential*. This measures how fast genetic evolution occurs for a given deviation of the mean phenotype from the optimum. The strength of stabilizing selection *γ* measures how the mean fitness decreases with the squared deviation from the optimum and also determines the strength of directional selection for a given deviation from the optimum. Stronger stabilizing selection (larger *γ*), although causing increased mortality, allows faster environmental change to be tolerated by causing faster evolution [13]. The genetic variance, the product of phenotypic variance *σ* ^2^ and heritability *h* ^2^, determines how much genetic evolution is caused by a given strength of selection. Higher genetic variance allows persistence under stronger environmental change, because it allows the population to track its phenotypic optimum more closely. Although we focus on a single trait for simplicity, this may actually be a linear combination of measurable traits. In this case, the genetic variances of the original traits and their genetic covariances affect the magnitude of the genetic response to selection in any phenotypic direction [24],[25].

(3) *Biological impact of the environment*. This links the environment to the biology of individuals in the species. Phenotypic plasticity describes the direct impact of the environment on the development of individual phenotypes. It may involve morphological, physiological or behavioural responses, which can occur on different time scales. For continuous environmental variables, plasticity usually is modelled using reaction norms, where the phenotype of a given genotype is plotted as a function of the environment [26]. We focus on linear reaction norms for simplicity, although reaction norms can be non-linear [27],[28] (see below). With linear reaction norms, the slope *b* quantifies the degree of plasticity. The environmental sensitivity of selection, *B*, measures how the optimum phenotype changes with the environment, which for simplicity we also assume is a linear relationship. With no cost of plasticity, populations with *b* closer to *B* are likely to persist under higher rates of environmental change.

## A Research Program

This model can be combined with environmental projections to ask whether future rates of environmental change will threaten the persistence of particular populations or species. Application of the model requires multiple steps, which previously have been undertaken in isolation or in combination, although all have rarely been completed together.

### Identify Ecologically Important Traits Affecting Population Growth

Ecological investigation in the field is needed to identify traits that potentially determine adaptation to a specific environment. For instance, for ectotherms such as insects and reptiles, thermal adaptation may occur not only through physiological traits governing energy metabolism, but also through behavioural and morphological traits involved in movement between shaded and sunny patches [29]. For many bird species, adaptation to global warming involves adjusting their breeding date so that reproduction coincides with a peak in prey density [30].

The strength (and direction) of natural selection *γ* has been estimated in many plant and animal taxa by regressing the fitness of individuals on their phenotypes [31],[32]. Most of these studies focus on relative fitness, which determines the evolutionary dynamics. However, absolute fitness and *r_max_* are needed to understand how phenotypic traits influence population dynamics. Analysis of natural selection has been extended to age- or stage-structured populations, allowing identification of parts of the life cycle where phenotypes most strongly influence demography [33]–[36].

### Estimate Genetic Variance and Plasticity

The question of whether genetic variation (*h* ^2^ *σ* ^2^) along the direction of natural selection limits the rate of evolution has motivated many studies. For instance, in the North American prairie plant *Chamaecrista fasciculate*, small genetic variance for a linear combination of traits has been predicted to limit the response to selection caused by climate change [25]. Similarly, it was suggested that lack of genetic variation for desiccation and cold resistance restricts the geographic range of several *Drosophila* species [37] and would limit the response to projected climate change in one of them [38]. Changes in the genetic and phenotypic variation with the environment may also be important. For example, the response to selection on body weight in wild Soay sheep is reduced because of lower heritability in environments where selection is stronger [39].

The importance of phenotypic plasticity for rapid evolution in response to directional environmental change has only recently been fully appreciated. Many authors have observed adaptive phenotypic evolution without being able to firmly attribute it to genetic change (reviewed in [40]), but relatively few studies have progressed from considering plasticity as a nuisance parameter to measuring norms of reaction, and their slopes *b*, in the context of climate change [41]. This was done recently for reproductive timing in mammals [42] and birds [43],[44].

### Measure the Environmental Sensitivity of Selection

Few investigators have measured the environmental sensitivity of phenotypic selection as described by parameter *B* in Box 2. The most relevant studies have measured selection in a small number of discrete environments. This approach has been applied to birds to describe the influence of changing seasonality on selection on breeding date [45], and in plants to examine the impact of drought on selection on physiological traits [46],[47]. It has also been combined with experimental manipulation of the environment, mainly in plants, using either abiotic [48] or biotic factors, such as crowding [49],[50] or natural enemies [51],[52]. Other studies have employed ecological criteria to identify changes in the optimum phenotype without actually measuring selection. For instance, the optimum egg-laying date for birds can be deduced from the temporal peak in food availability determined by the population dynamics of insects on which they feed [53].

What emerges from this brief outline is that two key areas of study are lacking: the quantification of the environmental sensitivity of selection across continuous environments and the detailed investigation of phenotypic plasticity, including its costs and limits under sustained environmental change. Further work, such as that outlined below, would begin to make predictive approaches possible.

## Phenotypic Selection along an Environmental Gradient

**Figure 1:**
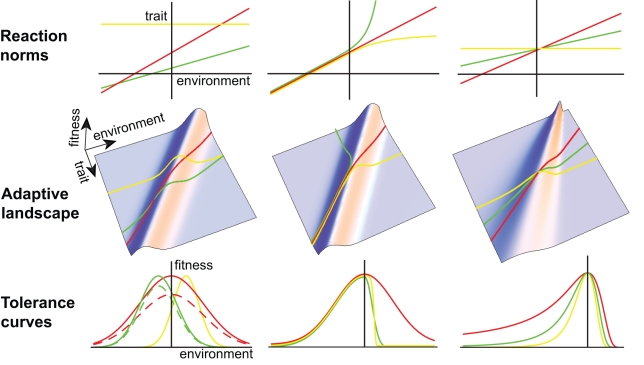
Tolerance curves and phenotypic plasticity. *First row*: norms of reaction for three genotypes (colored lines); *second row*: generalized adaptive landscape depicting fitness as a function of the phenotype and the environment, with the reaction norms projected up onto the fitness surface from the lower plane; *third row*: environmental tolerance curves representing slices through the adaptive landscape along the lines defined by the reaction norms. The three columns represent alternative scenarios described in the text. In the lower left panel, the dashed green and dashed red tolerance curves include costs of plasticity.

A relationship between an environmental variable and a measure of selection, either observed in the wild or derived from an experiment, has been argued to be the strongest evidence for the cause of natural selection [54]. Quantification of this relationship is required to predict population evolution and persistence in a changing environment. Most of the studies highlighted above compared selective pressures among discrete environmental states, often interpreted as stresses (but see [39]). Current global change, however, mainly involves continuous environmental variables for which precise time series and projections are available. The environmental sensitivity of selection should therefore be estimated by measuring phenotypic selection along a continuous environmental gradient. Apart from measuring a fundamental parameter of the model, this would also allow its basic assumptions to be tested. In particular, our model (like previous ones) assumes that environmental change primarily alters the optimum phenotype with little impact on the width of the fitness function (Figure 1), but this assumption has hardly ever been tested empirically.

Measurement of selection in several environments may be difficult to carry out in the field because of lack of control over environmental parameters. For some species such measurements could be performed in controlled laboratory conditions, provided the relationship between fitness in the laboratory and in the wild can be established. Alternatively, performance can be measured in the laboratory and the relationship between performance and fitness assessed in the wild [55].

Studies of thermal tolerance curves in ectotherms commonly measure fitness in continuous environments. However, these studies generally relate fitness (or performance) directly to the environment, which makes it difficult to produce evolutionary predictions and to test them experimentally [56]. Modelling tolerance curves with an underlying phenotypic trait subject to natural selection and phenotypic plasticity may prove more fruitful, since it should allow predictions about the dynamics of tolerance curve evolution (Box 3).

**Box 3. Tolerance Curves and Phenotypic Plasticity**

Environmental tolerance curves relating fitness (or performance) to abiotic environmental variables such as temperature [56] or salinity [77] have been studied for a wide variety of organisms, ranging from reptiles and amphibians to insects and plants [56]. They are generally characterized by a few descriptive variables, such as critical environments (where fitness or performance vanishes), optimum environment (where fitness is maximized), and skewness. Previous models investigated the benefits of broad versus narrow tolerance curves under several forms of environmental change [61],[62],[78], but they failed to produce predictions about the dynamics of and constrains on tolerance curve evolution. Recently there have been calls for new theoretical developments with clearer empirically testable outcomes [56].

We propose that incorporating phenotypically plastic traits, which are under stabilizing selection through their influence on lifetime fitness [33],[34],[79], can clarify how tolerance curves emerge and evolve (Figure 1). With linear reaction norms and constant strength of stabilizing selection across environments (ridge with constant width in the adaptive landscape), the reaction norm slope determines tolerance breadth, while the reaction norm intercept (or elevation) determines the optimum environment (first column in Figure 1). With genetic variation in the slope of reaction norms, the tolerance breadth may evolve as a consequence of evolving phenotypic plasticity in the underlying trait. Including a cost of plasticity, where fitness decreases with reaction norm slope regardless of the trait value [80],[81], produces a generalist–specialist trade-off between tolerance breadth and maximum fitness, corresponding to the intuitive idea that a “Jack-of-all-trades is a master of none.” This is illustrated in the bottom left panel of Figure 1—with cost an increasing function of plasticity (dashed lines), the red dashed tolerance curve has a lower maximum fitness than the green dashed curve, because the former reaction norm has greater plasticity. This approach can be used to make testable predictions in specific cases based on measures of the variability, inheritance, and plasticity of phenotypic traits.

Thermal tolerance curves generally are strongly skewed, with fitness decreasing steeply as temperature approaches the critical thermal maximum [56]. This could be caused by non-linear reaction norms for underlying traits, as illustrated by the second column in Figure 1. Plastic phenotypic responses that saturate (yellow genotype) or increase exponentially (green genotype) in extreme environments could both induce skewed tolerance curves. Alternatively, skewed tolerance curves could be caused by the strength of stabilizing selection (width of the fitness ridge) changing with the environment (third column in Figure 1). Finally, the fitness function acting on the trait may itself be skewed in all environments, the height of the fitness ridge may depend on the environment, or the optimum phenotype may change non-linearly with the environment (not shown). These five possible causes of skewness have different implications for the evolution of thermal tolerance in a changing environment, and need to be tested empirically.

## Costs and Limits of Plasticity in a Changing Environment

### Plasticity in Extreme Environments

The model in Box 2 assumes a linear reaction norm, implying that a given amount of environmental change always produces the same plastic phenotypic change. Plasticity is generally studied in the context of environments that vary in space or fluctuate in time with a stationary distribution, but little is known about plastic responses outside the usual range of variation. Extreme environments may disrupt the plastic response, such that the reaction norm may take any shape in environments that were rarely encountered before [57]. This argument is based on the theory of the evolution of reaction norm in heterogeneous environments, assuming little genetic constraint on reaction norm shape. However, reaction norm shapes are likely to be genetically constrained. For many traits, plastic phenotypic responses should reach a physiological limit and “saturate” in extreme environments [58]. For instance, body size or metabolic rate obviously cannot increase or decrease indefinitely under sustained environmental change. For other traits, disruption of homeostasis may cause the plastic response to be amplified in extreme environments. Both of these mechanisms could generate skewed tolerance curves like those observed for response to temperature, even when phenotypic selection is symmetric (Box 3).

### Costs of Plasticity and the Generalist versus Specialist Trade-Off

Phenotypic plasticity may entail several types of fitness costs to the organism independent of the expressed phenotype [59]. Here, we shall only distinguish two kinds of costs of plasticity: constitutive and induced. A constitutive cost reduces individual fitness depending only on the degree of plasticity. This includes the cost of maintaining physiological machinery that allows phenotypic plasticity or acquiring information about the environment [59]. The model in Box 2 focuses on such costs. In contrast an induced cost is a reduction in fitness that depends on the amount of plastic phenotypic change. This kind of cost is physiological and can be understood in terms of constraints on energy allocation. It applies mainly to labile traits that may change repeatedly during the lifetime of an individual, such as breeding date for birds [60].

In the literature on tolerance curves, the cost of plasticity is generally expressed as a trade-off between tolerance breadth and fitness in the optimum environment, corresponding to the intuitive idea that the “Jack-of-all-trades is a master of none.” This has been modelled [61],[62], but the empirical evidence is still controversial [56],[63] and based mainly on comparative data among taxa rather than on within-species variation (but see [64]). We propose that this trade-off may be a consequence of the cost of plasticity for an underlying phenotypic trait (Box 3, Figure 1).

**Figure 2:**
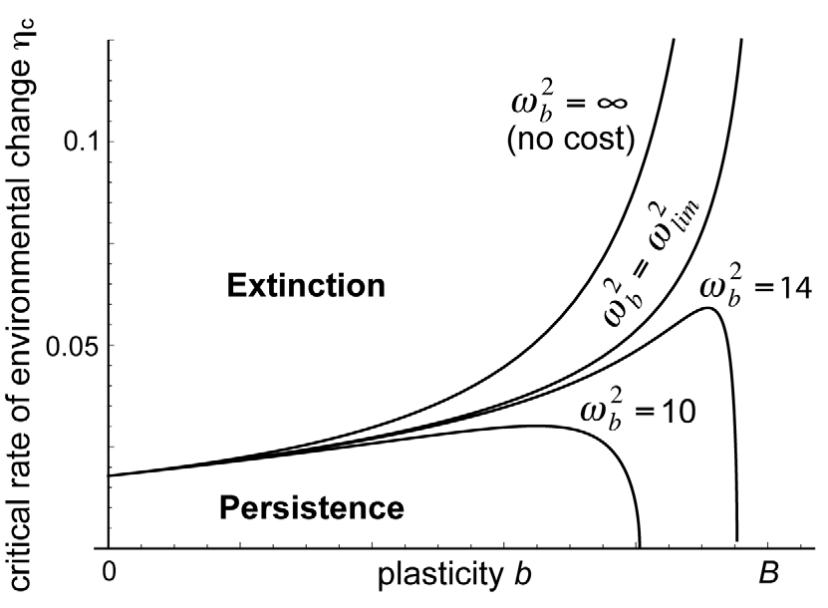
Critical rate of environmental change with costly phenotypic plasticity. The maximum rate of environmental change allowing long-term persistence of a population, *η_c_*, is plotted against plasticity *b*, for several values of the cost of plasticity. For a given plasticity *b*, the cost increases with decreasing *ω_b_*. For each *ω_b_*, rates of environmental change higher than the corresponding line cause population extinction. Parameters: *r_max_* = 0.140, *T* = 1, *γ* = 1/51, *B* = 2, *σ* ^2^ = 1, and *h* ^2^ = 0.5.

In the model of Box 2 and Figure 2, we show how a constitutive cost of plasticity can limit the critical rate of environmental change for population persistence. An induced cost would yield a similar result in this model since the environment changes at a constant rate. The cost of plasticity may thus limit population persistence in a changing environment, yet little is known about its importance in natural populations. A recent review showed that such costs may be widespread but weak [65]. Further research is needed to measure costs of plasticity for organisms with long generations (such as large mammals and trees) or with life cycles that depend on seasonal timing (e.g., interaction of temperature and photoperiod [66]), which rely most on plasticity to persist in a rapidly changing environment.

## Limitations of the Model

Our model includes a number of simplifying assumptions. We assume a constant shape of the fitness function across environments, allowing only the optimum phenotype to change with the environment. However, the strength of stabilizing selection around the optimum phenotype *γ* may depend on the environment (Figure 1, third column, second row). Furthermore the maximum fitness *r_max_* may generally be lower in more extreme environments because of physiological constraints, such that the ridge in Figure 1 would have an intermediate maximum. This was shown to occur in a recent comparative study of thermal tolerance curves among several insect taxa, where the maximum fitness was lower for species that had their optimum at a lower temperature [67]. Other demographic parameters including the generation time *T* may change with the environment. Genotype × environment interaction (or genetic variance in plasticity) can cause the genetic variance (*h* ^2^ *σ* ^2^) to depend on the environment [68]–[71]. Although these mechanisms are not included in our simple model, they all can be analyzed theoretically and studied empirically, and should be included where they are suspected to be important.

Plasticity itself may evolve if it varies genetically, and new environments can cause directional selection on plasticity [72],[73]. However, more information is needed about plasticity and its inheritance in extreme environments before the evolution of plasticity can be included in the analysis of persistence under sustained environmental change.

Finally, we considered a population that cannot disperse nor receive migrants from other populations experiencing different environments. We focused on this situation because it is one that is most commonly overlooked in niche modelling studies. Our model thus applies best to species with habitats restricted by dispersal barriers or species which disperse slowly relative to the rate of environmental change [74]. At the other extreme, a population may be able to persist by following its preferred environment in space without any evolution, as assumed in niche modelling, but this is very unlikely for species occupying fragmented habitats. For populations that can both disperse and evolve in response to a changing environment, gene flow among populations with different environments may limit local adaptation and restrict the geographic range of the population or even cause extinction [74],[75].

## Conclusion

Our aim was to describe an approach based on evolutionary and demographic mechanisms that can be used to make predictions on population persistence in a changing environment and to highlight the most important variables to measure. While this approach is obviously more costly and time-consuming than niche modelling, its results are also likely to be more useful for specific purposes because it explicitly incorporates the factors that limit population response to environmental change.

The feasibility of such a mechanistic approach has been demonstrated by a few recent studies. Deutsch et al. [76] used thermal tolerance curves to predict the fitness consequence of climate change for many species of terrestrial insects across latitudes, but without explicitly considering phenotypic plasticity or genetic evolution. Kearney et al. [12] combined biophysical models of energy transfers with measures of heritability of egg desiccation to predict how climate change would affect the distribution of the mosquito *Aedes aegiptii* in Australia. Egg desiccation was treated as a threshold trait, but the possibility of phenotypic plasticity or evolution of the threshold was not considered. These encouraging efforts call for more empirical studies where genetic evolution and phenotypic plasticity are combined with demography to make predictions about population persistence in a changing environment. The simple approach we have outlined is a necessary step towards a more specific and comprehensive understanding of the influence of environmental change on population extinction.

## Supporting Information

Text S1 **A model of plasticity, evolution, and extinction in a changing environment.**
(0.48 MB PDF)

## References

[1] Peters R. L, Lovejoy T. E (1994). Global warming and biological diversity..

[2] Hutchinson G (1957). Concluding remarks.. Cold Springs Harbor Symp Quant Biol.

[3] Thomas C. D, Cameron A, Green R. E, Bakkenes M, Beaumont L. J (2004). Extinction risk from climate change.. Nature.

[4] Jackson S, Betancourt J, Booth R, Gray S (2009). Ecology and the ratchet of events: climate variability, niche dimensions, and species distributions.. Proc Natl Acad Sci U S A.

[5] Araujo M, Pearson R (2005). Equilibrium of species' distributions with climate.. Ecography.

[6] Guisan A, Thuiller W (2005). Predicting species distribution: offering more than simple habitat models.. Ecol Lett.

[7] Randin C. F, Engler R, Normand S, Zappa M, Zimmermann N. E (2009). Climate change and plant distribution: local models predict high-elevation persistence.. Glob Change Biol.

[8] Pulliam H. R (2000). On the relationship between niche and distribution.. Ecology Letters.

[9] Crozier L, Dwyer G (2006). Combining population-dynamic and ecophysiological models to predict climate-induced insect range shifts.. Am Nat.

[10] Kearney M, Porter W (2009). Mechanistic niche modelling: combining physiological and spatial data to predict species' ranges.. Ecol Lett.

[11] Morin X, Viner D, Chuine I (2008). Tree species range shifts at a continental scale: new predictive insights from a process-based model.. J Ecol.

[12] Kearney M, Porter W. P, Williams C, Ritchie S, Hoffmann A. A (2009). Integrating biophysical models and evolutionary theory to predict climatic impacts on species' ranges: the dengue mosquito *Aedes aegypti* in Australia.. Funct Ecol.

[13] Lynch M, Lande R, Kareiva P, Kingsolver J, Huey R (1993). Evolution and extinction in response to environmental change.. Biotic interactions and global change.

[14] Burger R, Lynch M (1995). Evolution and extinction in a changing environment - a quantitative-genetic analysis.. Evolution.

[15] Willi Y, Hoffmann A. A (2009). Demographic factors and genetic variation influence population persistence under environmental change.. J Evol Biol.

[16] Solomon S, Qin D, Manning M, Chen Z, Marquis M (2008). Climate change 2007: the physical science basis..

[17] Davis M. B, Shaw R. G, Etterson J. R (2005). Evolutionary responses to changing climate.. Ecology.

[18] Jump A, Penuelas J (2005). Running to stand still: adaptation and the response of plants to rapid climate change.. Ecol Lett.

[19] Lande R (1988). Genetics and demography in biological conservation.. Science.

[20] Gomulkiewicz R, Houle D (2009). Demographic and genetic constraints on evolution.. Am Nat.

[21] Blueweiss L, Fox H, Kudzma V, Nakashima D, Peters R (1978). Relationships between body size and some life history parameters.. Oecologia.

[22] Charnov E (1993). Life history invariants: some explorations of symmetry in evolutionary ecology..

[23] Fenchel T (1974). Intrinsic rate of natural increase: the relationship with body size.. Oecologia.

[24] Hansen T. F, Houle D (2008). Measuring and comparing evolvability and constraint in multivariate characters.. J Evol Biol.

[25] Etterson J. R, Shaw R. G (2001). Constraint to adaptive evolution in response to global warming.. Science.

[26] Scheiner S (1993). Genetics and evolution of phenotypic plasticity.. Annu Rev Ecol Syst.

[27] Gomulkiewicz R, Kirkpatrick M (1992). Quantitative genetics and the evolution of reaction norms.. Evolution.

[28] Kingsolver J. G, Ragland G. J, Shlichta J. G (2004). Quantitative genetics of continuous reaction norms: thermal sensitivity of caterpillar growth rates.. Evolution.

[29] Kearney M, Shine R, Porter W. P (2009). The potential for behavioral thermoregulation to buffer “cold-blooded” animals against climate warming.. Proc Natl Acad Sci U S A.

[30] Visser M. E (2008). Keeping up with a warming world; assessing the rate of adaptation to climate change.. Proc R Soc Lond B Biol Sci.

[31] Lande R, Arnold S. J (1983). The measurement of selection on correlated characters.. Evolution.

[32] Kingsolver J. G, Hoekstra H. E, Hoekstra J. M, Berrigan D, Vignieri S. N (2001). The strength of phenotypic selection in natural populations.. Am Nat.

[33] Lande R (1982). A quantitative genetic theory of life-history evolution.. Ecology.

[34] van Tienderen P (2000). Elasticities and the link between demographic and evolutionary dynamics.. Ecology.

[35] Coulson T, Tuljapurkar S (2008). The dynamics of a quantitative trait in an age-structured population living in a variable environment.. Am Nat.

[36] Ozgul A, Tuljapurkar S, Benton T. G, Pemberton J. M, Clutton-Brock T. H (2009). The dynamics of phenotypic change and the shrinking sheep of St. Kilda.. Science.

[37] Kellermann V, van Heerwaarden B, Sgro C. M, Hoffmann A. A (2009). Fundamental evolutionary limits in ecological traits drive *Drosophila* species distributions.. Science.

[38] Hoffmann A. A, Hallas R. J, Dean J. A, Schiffer M (2003). Low potential for climatic stress adaptation in a rainforest *Drosophila* species.. Science.

[39] Wilson A. J, Pemberton J. M, Pilkington J. G, Coltman D. W, Mifsud D. V (2006). Environmental coupling of selection and heritability limits evolution.. PLoS Biol.

[40] Hendry A. P, Farrugia T. J, Kinnison M. T (2008). Human influences on rates of phenotypic change in wild animal populations.. Mol Ecol.

[41] Gienapp P, Teplitsky C, Alho J. S, Mills J. A, Merila J (2008). Climate change and evolution: disentangling environmental and genetic responses.. Mol Ecol.

[42] Reale D, McAdam A. G, Boutin S, Berteaux D (2003). Genetic and plastic responses of a northern mammal to climate change.. Proc R Soc Lond B Biol Sci.

[43] Charmantier A, McCleery R. H, Cole L. R, Perrins C, Kruuk L. E (2008). Adaptive phenotypic plasticity in response to climate change in a wild bird population.. Science.

[44] Nussey D. H, Postma E, Gienapp P, Visser M. E (2005). Selection on heritable phenotypic plasticity in a wild bird population.. Science.

[45] Both C, Visser M. E (2001). Adjustment to climate change is constrained by arrival date in a long-distance migrant bird.. Nature.

[46] Dudley S. A (1996). Differing selection on plant physiological traits in response to environmental water availability: a test of adaptive hypotheses.. Evolution.

[47] Franke D. M, Ellis A. G, Dharjwa M, Freshwater M, Fujikawa M (2006). A steep cline in flowering time for *Brassica rapa* in southern California: population-level variation in the field and the greenhouse.. Int J Plant Sci.

[48] Stanton M. L, Roy B. A, Thiede D. A (2000). Evolution in stressful environments. I. Phenotypic variability, phenotypic selection, and response to selection in five distinct environmental stresses.. Evolution.

[49] Weinig C (2000). Differing selection in alternative competitive environments: shade-avoidance responses and germination timing.. Evolution.

[50] Donohue K, Messiqua D, Pyle E. H, Heschel M. S, Schmitt J (2000). Evidence of adaptive divergence in plasticity: density- and site-dependent selection on shade-avoidance responses in *Impatiens capensis*.. Evolution.

[51] Gomez J. M (2003). Herbivory reduces the strength of pollinator-mediated selection in the Mediterranean herb *Erysimum mediohispanicum*: consequences for plant specialization.. Am Nat.

[52] Mauricio R, Rausher M. D (1997). Experimental manipulation of putative selective agents provides evidence for the role of natural enemies in the evolution of plant defense.. Evolution.

[53] van Asch M, van Tienderen P. H, Holleman L. J. M, Visser M. E (2007). Predicting adaptation of phenology in response to climate change, an insect herbivore example.. Glob Change Biol.

[54] Wade M. J, Kalisz S (1990). The causes of natural selection.. Evolution.

[55] Arnold S (1983). Morphology, performance and fitness.. Integr Compar Biol.

[56] Angilletta M (2009). Thermal adaptation: a theoretical and empirical synthesis..

[57] Ghalambor C, McKay J, Carroll S, Reznick D (2007). Adaptive versus non-adaptive phenotypic plasticity and the potential for contemporary adaptation in new environments.. Funct Ecol.

[58] Rocha F, Medeiros H. F, Klaczko L. B (2009). The reaction norm for abdominal pigmentation and its curve in *Drosophila mediopunctata* depend on the mean phenotypic value.. Evolution.

[59] Dewitt T. J, Sih A, Wilson D. S (1998). Costs and limits of phenotypic plasticity.. Trends Ecol Evol.

[60] Nussey D. H, Wilson A. J, Brommer J. E (2007). The evolutionary ecology of individual phenotypic plasticity in wild populations.. J Evol Biol.

[61] Gilchrist G. W (1995). Specialists and generalists in changing environments .1. Fitness landscapes of thermal sensitivity.. Am Nat.

[62] Lynch M, Gabriel W (1987). Environmental tolerance.. Am Nat.

[63] Huey R, Hertz P (1984). Is a Jack-of-all-temperatures a master of none?. Evolution.

[64] Gilchrist G. W (1996). A quantitative genetic analysis of thermal sensitivity in the locomotor performance curve of *Aphidius ervi*.. Evolution.

[65] Van Buskirk J, Steiner U. K (2009). The fitness costs of developmental canalization and plasticity.. J Evol Biol.

[66] Bradshaw W. E, Holzapfel C. M (2008). Genetic response to rapid climate change: it's seasonal timing that matters.. Mol Ecol.

[67] Frazier M. R, Huey R. B, Berrigan D (2006). Thermodynamics constrains the evolution of insect population growth rates: “warmer is better”.. Am Nat.

[68] Via S, Lande R (1985). Genotype-environment interaction and the evolution of phenotypic plasticity.. Evolution.

[69] Falconer D. S, Mackay T. F (1996). Introduction to quantitative genetics..

[70] Hoffmann A. A, Merila J (1999). Heritable variation and evolution under favourable and unfavourable conditions.. Trends Ecol Evol.

[71] Charmantier A, Garant D (2005). Environmental quality and evolutionary potential: lessons from wild populations.. Proc R Soc Lond B Biol Sci.

[72] Gavrilets S, Scheiner S. M (1993). The genetics of phenotypic plasticity .6. Theoretical predictions for directional selection.. J Evol Biol.

[73] Lande R (2009). Adaptation to an extraordinary environment by evolution of phenotypic plasticity and genetic assimilation.. J Evol Biol.

[74] Polechova J, Barton N, Marion G (2009). Species' range: adaptation in space and time.. Am Nat.

[75] Pease C. M, Lande R, Bull J. J (1989). A model of population-growth, dispersal and evolution in a changing environment.. Ecology.

[76] Deutsch C. A, Tewksbury J. J, Huey R. B, Sheldon K. S, Ghalambor C. K (2008). Impacts of climate warming on terrestrial ectotherms across latitude.. Proc Natl Acad Sci U S A.

[77] Browne R. A, Wanigasekera G (2000). Combined effects of salinity and temperature on survival and reproduction of five species of Artemia.. J Exp Mar Biol Ecol.

[78] Huey R. B, Kingsolver J. G (1993). Evolution of resistance to high temperature in ectotherms.. Am Nat.

[79] Charlesworth B (1994). Evolution in age-structured populations..

[80] Van Tienderen P (1991). Evolution of generalists and specialists in spatially heterogeneous environments.. Evolution.

[81] Chevin L. M, Lande R (2010). When do adaptive plasticity and genetic evolution prevent extinction of a density-regulated population?. Evolution.

