# Supplementary material for: Adaptation, Plasticity, and Extinction in a Changing Environment: Towards a Predictive Theory
Source: PLoS Biol. 2010 Apr 27;8(4):e1000357. doi: 10.1371/journal.pbio.1000357 (PMC2864732; doi:10.1371/journal.pbio.1000357)
Supplement: Text S1 — A model of plasticity, evolution, and extinction in a changing environment. (0.48 MB PDF) [file pbio.1000357.s001.pdf]

Text S1. A model of plasticity, evolution and extinction in a changing environment

We model the evolution and growth of a population in a constantly changing environment as in Lynch and Lande's model [1], but with partially adaptive phenotypic plasticity. Time *t* is measured in arbitrary units (e.g., years or days), and we assume discrete non-overlapping generations, with generation time *T*. An environmental variable ε changes at a constant rate in time, $\varepsilon = \eta t$. Adaptation to the changing environment is mediated by a quantitative trait *z*, which exhibits phenotypic plasticity modelled using linear reaction norms. We focus on a plastic trait with determinate growth, for which the adult phenotype is determined by the environment at a critical stage of development a fraction τ of a generation before selection acting only on adults [2,3]. The phenotype of an adult just before selection at generation *n* is $z = a + b\eta[T(n-\tau)] + e$. Reaction norm elevation *a* is the breeding value of a genotype in an arbitrary reference environment (chosen as 0 without loss of generality), and the slope *b* quantifies its plasticity. The residual component of phenotypic variation *e* is assumed to be normally distributed with mean 0 and constant variance σ_e^2. We also suppose that breeding values are normally distributed with constant variance σ_a^2, but that plasticity has no genetic variance, so *b* is constant and cannot evolve. The phenotype distribution *p*(*z*) is thus normal with mean $\bar{z}$ and constant variance $\sigma^2 = \sigma_a^2 + \sigma_e^2$. The mean phenotype before selection at generation *n* is $\bar{z} = \bar{a} + b\eta[T(n-\tau)]$.

Plasticity is assumed to entail a fitness cost, regardless of the expressed value of the trait. This is modelled using Gaussian stabilizing selection towards

lower absolute plasticity, as in previous models [4,5]. Apart (and independent) from the cost of plasticity, the phenotypic trait is also under Gaussian stabilizing selection for an optimum θ that changes linearly with the environment, $\theta = B\varepsilon$, where B is the environmental sensitivity of phenotypic selection. The expected lifetime fitness of individuals with phenotype z is thus

$$W(z) = W_{max} \exp\left\{ -\frac{(z-\theta)^2}{2\omega_z^2} - \frac{b^2}{2\omega_b^2} \right\}, \qquad \text{(A1)}$$

where ω_z^2 and ω_b^2 are the widths of the fitness functions on the trait and on plasticity, respectively. We focus on partially adaptive plasticity, that is on cases where $0 < b < B$.

We assume density-independent population growth, such that the recursion for the population size over one generation is $N' = \overline{W}N$, where $\overline{W} = \int W(z)p(z)dz$ is the mean fitness in the population at generation n and N' is the population size at generation n+1. The population is decreasing in size if $\overline{W} < 1$ (or $\ln\overline{W} < 0$). At generation n, the growth rate of log population size per unit time is

$$r = \frac{\ln\overline{W}}{T} = r_{max} - \frac{\gamma(\bar{z}-\theta)^2}{2T} \qquad \text{(A2a)}$$

where $\gamma = 1/(\omega_z^2 + \sigma^2)$ is the strength of stabilizing selection and

$$r_{max} = \frac{\ln W_{max}}{T} - \frac{\ln\left(1 + \sigma^2/\omega_z^2\right) + b^2/\omega_b^2}{2T} \qquad \text{(A2b)}$$

is the maximum growth rate of the population when the mean phenotype is at the optimum. This growth rate is reduced both by the phenotypic variance of the trait and by the cost of plasticity.

The change in the mean phenotype per unit time is

$$\frac{\Delta \bar{z}}{T} = \frac{\Delta \bar{a} + b\eta T}{T}, \tag{A3}$$

where $\Delta \bar{a}$ is the per-generation change in the mean breeding value caused by natural selection on the trait, and $b\eta T$ is the plastic phenotypic change between generations. The genetic change in mean phenotype per generation is the product of the selection gradient and the genetic variance of the trait [6],

$$\Delta \bar{a} = \frac{\partial \ln \overline{W}}{\partial \bar{a}} \sigma_a^2 = -\gamma(\bar{z} - \theta)\sigma_a^2. \tag{A4}$$

Partially adaptive plasticity b causes the mean phenotype to be closer to the optimum (since $\bar{z} = \bar{a} + b\eta T(n - \tau)$ and $\theta = B\eta T n$), thus reducing the strength of directional selection on the trait and thereby also reducing the genetic response to selection. However the smaller genetic change in the trait is counterbalanced by the plastic phenotypic response in eq. (A3).

With the mean phenotypic initially at the optimum, it can be shown that the deviation of the mean phenotype from the optimum increases before reaching a constant value (phenotypic lag). The magnitude of the phenotypic lag determines population growth rate and whether the population can persist in the changing environment [1]. The stationary state of the system occurs when the rate of change in the trait equals the rate of change in the optimum phenotype, $\Delta \bar{z} / T = B\eta$. Combining this condition with equations (A3) and (A4) yields the equilibrium phenotypic lag

$$(\theta - \bar{z})_{eq} = \frac{(B - b)\eta T}{\gamma \sigma_a^2} \tag{A5}$$

and (from eq. A2) to the equilibrium growth rate of the population

$$r_{eq} = r_{\max} - \frac{(B - b)^2 \eta^2 T}{2\gamma \sigma_a^4}. \tag{A6}$$

Solving r_{eq} = 0 for η produces eq. (1) in Box 1 for the critical rate of environmental change η_c with constant phenotypic plasticity.

The cost of plasticity and the plastic phenotypic response have opposing effects on the critical rate of environmental change η_c. The critical rate η_c is maximized for an intermediate value of $\alpha = b/B$, the ratio of phenotypic plasticity to the environmental sensitivity of selection. From eqs. (A2b) and (A3), and assuming weak selection ($\omega_z^2 >> \sigma^2$), the optimum relative plasticity is

$$\begin{cases} \alpha_o = \dfrac{s_{lim}}{s_b} & \text{if } s_b > s_{lim} \\ \alpha_o = 1 & \text{if } s_b \le s_{lim} \end{cases} \tag{A7}$$

where $s_b = B^2/(2\omega_b^2)$ is a scaled intensity of the cost of plasticity, and $s_{lim} = \ln W_{max} - (\gamma\sigma^2)/2$ is the threshold for this scaled cost of plasticity above which perfect plasticity (α = 1) is optimal.

References

1. Lynch M, Lande R (1993) Evolution and extinction in response to environmental change. In: Kareiva P, Kingsolver J, Huey R, editors. Biotic interactions and global change. Sunderland, Ma: Sinauer. pp. 234-250.
2. de Jong G (1999) Unpredictable selection in a structured population leads to local genetic differentiation in evolved reaction norms. J Evol Biol 12: 839-851.
3. Lande R (2009) Adaptation to an extraordinary environment by evolution of phenotypic plasticity and genetic assimilation. J Evol Biol 22: 1435-1446.
4. van Tienderen P (1991) Evolution of generalists and specialists in spatially heterogeneous environments. Evolution 45: 1317-1331.
5. Chevin LM, Lande R (2010) When do adaptive plasticity and genetic evolution prevent extinction of a density-regulated population? Evolution (*in press*).
6. Lande R (1976) Natural selection and random genetic drift in phenotypic evolution. Evolution 30: 314-334.
